# Phase recovery and holographic image reconstruction using deep learning in neural networks

**DOI:** 10.1038/lsa.2017.141

**Published:** 2018-02-23

**Authors:** Yair Rivenson, Yibo Zhang, Harun Günaydın, Da Teng, Aydogan Ozcan

**Affiliations:** 1 Electrical and Computer Engineering Department, University of California, Los Angeles, CA 90095, USA; 2 Bioengineering Department, University of California, Los Angeles, CA 90095, USA; 3 California NanoSystems Institute (CNSI), University of California, Los Angeles, CA 90095, USA; 4 Computer Science Department, University of California, Los Angeles, CA 90095, USA; 5 Department of Surgery, David Geffen School of Medicine, University of California, Los Angeles, CA 90095, USA

**Keywords:** deep learning, holography, machine learning, neural networks, phase recovery

## Abstract

Phase recovery from intensity-only measurements forms the heart of coherent imaging techniques and holography. In this study, we demonstrate that a neural network can learn to perform phase recovery and holographic image reconstruction after appropriate training. This deep learning-based approach provides an entirely new framework to conduct holographic imaging by rapidly eliminating twin-image and self-interference-related spatial artifacts. This neural network-based method is fast to compute and reconstructs phase and amplitude images of the objects using only one hologram, requiring fewer measurements in addition to being computationally faster. We validated this method by reconstructing the phase and amplitude images of various samples, including blood and Pap smears and tissue sections. These results highlight that challenging problems in imaging science can be overcome through machine learning, providing new avenues to design powerful computational imaging systems.

## Introduction

Optoelectronic sensor arrays, such as charge-coupled devices (CCDs) or complementary metal-oxide-semiconductor (CMOS)-based imagers, are only sensitive to the intensity of light; therefore, phase information of the objects or the diffracted light waves cannot be directly recorded using such imagers. Phase recovery from intensity-only measurements has emerged as an important field to recover this lost phase information in the detection process, enabling the reconstruction of the phase and amplitude images of specimen using various approaches^1, 2, 3, 4, 5, 6, 7, 8, 9, 10, 11, 12, 13^. In fact, Gabor’s original in-line holography system^14^, where the diffracted light from the object interferes with the background light that is directly transmitted, is an important example where phase recovery is required to separate the twin-image and self-interference-related spatial artifacts from the real image of the sample. In various implementations, to improve the performance of the phase recovery and image reconstruction processes, additional intensity information is recorded, for example, by scanning the illumination source aperture^15, 16, 17, 18^, sample-to-sensor distance^19, 20, 21, 22, 23^ (in some cases referred to as out-of-focus imaging^24^), wavelength of illumination^25, 26^, or phase front of the reference beam^27, 28, 29, 30^, among other methods^31, 32, 33, 34, 35, 36^. All these methods utilize additional physical constraints and intensity measurements to robustly retrieve the missing phase information based on an analytical and/or iterative solution that satisfies the wave equation. Some of these phase retrieval techniques have enabled discoveries in different fields^37, 38, 39, 40^.

In this paper, we report a convolutional neural network-based method, trained through deep learning^41, 42^, that can perform phase recovery and holographic image reconstruction using a single hologram intensity. Deep learning is a machine learning technique that uses a multi-layered artificial neural network for data modeling, analysis and decision making and has shown considerable success in areas where large amounts of data are available. Deep learning has recently been applied to solving inverse problems in imaging science such as in super-resolution^43, 44^, acceleration of the image acquisition speed of computed tomography (CT)^45^, magnetic resonance imaging (MRI)^46^, photoacoustic tomography^47^ and holography^48, 49^.

In this work, we used deep learning to rapidly perform phase recovery and reconstruct complex-valued images of specimen using a single intensity-only hologram. This process is very fast, requiring approximately 3.11 s on a graphics processing unit (GPU)-based laptop computer to recover the phase and amplitude images of a specimen over a field of view of 1 mm^2^ with ~7.3 megapixels in each image channel (amplitude and phase). We validated this approach by reconstructing the complex-valued images of various samples, such as blood and Papanicolaou (Pap) smears as well as thin sections of human tissue samples, all of which demonstrated successful elimination of the twin-image and self-interference-related spatial artifacts that arise due to lost phase information during the hologram detection process. In other words, the convolutional neural network, after its training, learned to extract and separate the spatial features of the real image from the features of the twin-image and other undesired interference terms for both the phase and amplitude channels of the object. Remarkably, this deep learning-based phase recovery and holographic image reconstruction approach has been achieved without any modeling of light–matter interaction or wave interference. However, this does not imply that the presented approach entirely ignores the physics of light–matter interaction and holographic imaging, which is in fact statistically inferred through deep learning in the convolutional neural network by using a large number of microscopic images as the gold standard in the training phase. This training and statistical optimization of the neural network is performed once and can be considered as part of a blind reconstruction framework that performs phase recovery and holographic image reconstruction using a single input such as an intensity-only hologram of the object. This framework introduces a myriad of opportunities to design fundamentally new coherent imaging systems and can be broadly applicable to any phase recovery problem, spanning different parts of the electromagnetic spectrum, including visible wavelengths as well as X-rays^28, 30, 50, 51^.

## Results and discussion

**Figure 1:**
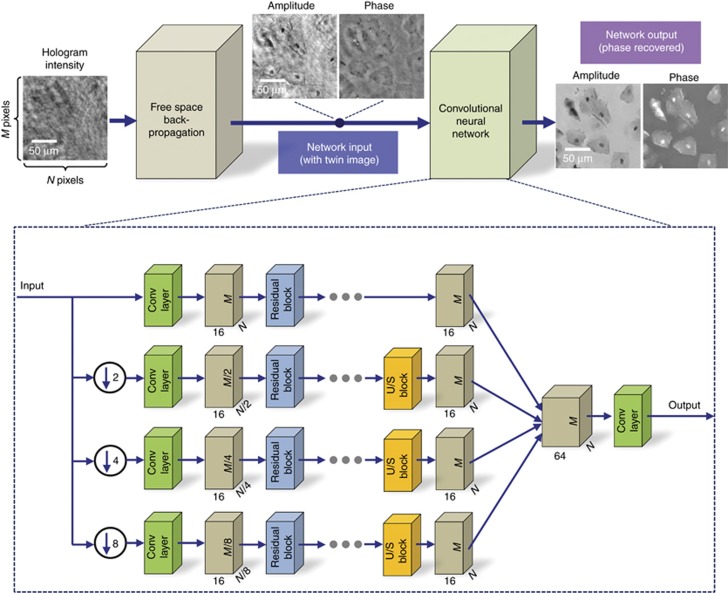
Following its training phase, the deep neural network blindly outputs artifact-free phase and amplitude images of the object using only one hologram intensity. This deep neural network is composed of convolutional layers, residual blocks and upsampling blocks (see Supplementary Information for additional details) and rapidly processes a complex-valued input image in a parallel, multi-scale manner.

**Figure 2:**
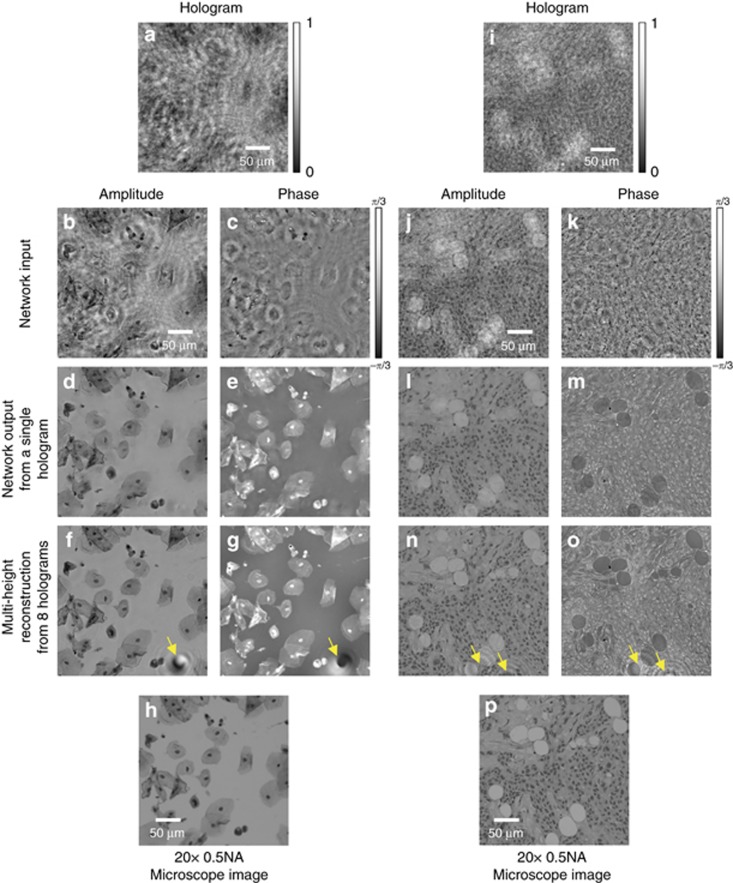
Comparison of the holographic reconstruction results for different types of samples: (**a-h**) Pap smear, (**i**-**p**) breast tissue section. (**a**, **i**) Zoomed-in regions of interest from the acquired holograms. (**b, c, j**, **k**) Amplitude and phase images resulting from free-space back-propagation of a single hologram intensity, shown in **a** and **i**, respectively. These images are contaminated with twin-image and self-interference-related spatial artifacts due to the missing phase information in the hologram detection process. (**d**, **e**, **l**, **m**) Corresponding amplitude and phase images of the same samples obtained by the deep neural network, demonstrating the blind recovery of the complex object image without twin-image and self-interference artifacts using a single hologram. (**f**, **g**, **n**, **o**) amplitude and phase images of the same samples reconstructed using multi-height phase retrieval with 8 holograms acquired at different sample-to-sensor distances. (**h**, **p**) corresponding bright-field microscopy images of the same samples, shown for comparison. The yellow arrows point to artifacts in **f**, **g, n, o** (due to out-of-focus dust particles or other unwanted objects) that are significantly suppressed by the network reconstruction, as shown in **d**, **e**, **l**, **m**.

Our deep neural network approach for phase retrieval and holographic image reconstruction is schematically described in Figure 1 (see also Supplementary Figs. S1–S4). In this work, we chose to demonstrate the proposed framework using lens-free digital in-line holography of transmissive samples, including human tissue sections and blood and Pap smears (see Matrials and Methods). Due to the dense and connected nature of these samples that we imaged, their holographic in-line imaging requires the acquisition of multiple holograms for accurate and artifact-free object recovery^52^. A schematic of our experimental setup is shown in Supplementary Fig. S5, where the sample is positioned very close to a CMOS sensor chip with a <1 mm sample-to-sensor distance, which provides an important advantage in terms of the sample field of view that can be imaged. However, due to this relatively short sample-to-sensor distance, the twin-image artifact of the in-line holography, which is a result of the lost phase information, is strong and severely obstructs the spatial features of the sample in both the amplitude and phase channels, as illustrated in Figures 1 and 2.

**Figure 3:**
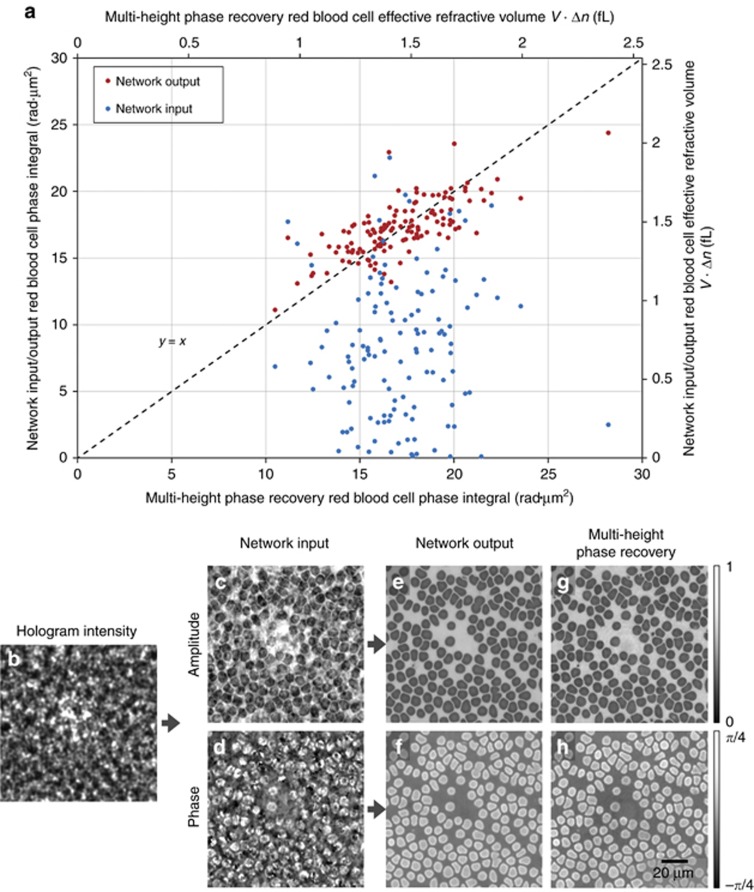
Red blood cell volume estimation using our deep neural network-based phase retrieval. The deep neural network output (**e**, **f**), given the input (**c**, **d**) obtained from a single hologram intensity (**b**), shows a good match with the multi-height phase recovery-based cell volume estimation results (**a**), calculated using *N*_holo_=8 (**g**, **h**). Similar to the yellow arrows shown in Figure 2f, 2g, 2n and 2o, the multi-height phase recovery results exhibit an out-of-focus fringe artifact at the center of the field-of-view in (**g, h**). Refer to Supplementary Information for the calculation of the effective refractive volume of cells.

The first step in our deep learning-based phase retrieval and holographic image reconstruction framework consists of ‘training’ the neural network. This training involves learning the statistical transformation between a complex-valued image that results from the back-propagation of a single intensity-only hologram of the object and the same object’s image that is reconstructed using a multi-height phase retrieval algorithm (treated as the gold standard for the training phase). This algorithm uses 8 hologram intensities acquired at different sample-to-sensor distances (see Materials and Methods as well as Supplementary Information). As illustrated in Figures 1,2,3, a simple back-propagation of the object’s hologram, without phase retrieval, contains severe twin-image and self-interference-related artifacts, hiding the phase and amplitude information of the object. This training/learning process (which is performed only once) results in a fixed deep neural network that is used to blindly reconstruct the phase and amplitude images of any object, free from twin-image and other undesired interference-related artifacts, using a single hologram intensity.

In our holographic imaging experiments, we used three different types of samples: blood smears, Pap smears and breast tissue sections, and separately trained three convolutional neural networks for each sample type, although the network architecture was identical in each case, as shown in Figure 1. To avoid over-fitting the neural network, we stopped the training when the deep neural network performance on the validation image set (which is different from the training image set and the blind testing image set) began to decline. We also accordingly made the network compact and applied pooling approaches^53^. Following this training process, each deep neural network was blindly tested with different objects that were not used in the training or validation image sets. Figures 1,2 and 3 show the neural network-based blind reconstruction results for the Pap smears, breast tissue sections and blood smears. These reconstructed phase and amplitude images clearly demonstrate the success of our deep neural network-based holographic image reconstruction approach to blindly infer artifact-free phase and amplitude images of the objects, matching the performance of the multi-height phase recovery. Table 1 further compares the structural similarity^54^ (SSIM) of our neural network output images (using a single input hologram, that is, *N*_holo_=1) against the results obtained with a traditional multi-height phase retrieval algorithm using multiple holograms (that is, *N*_holo_=2, 3,…,8) acquired at different sample-to-sensor distances. A comparison of the SSIM index values reported in Table 1 suggests that the imaging performance of the deep neural network using a single hologram is comparable to that of multi-height phase retrieval, closely matching the SSIM performance of *N*_holo_=2 for both Pap smear and breast tissue samples and the SSIM performance of *N*_holo_=3 for blood smear samples. The deep neural network-based reconstruction approach reduces the number of holograms required by 2-3 times. In addition to this reduction in the number of holograms, the computation time for holographic reconstruction using a neural network is also improved by more than three- and four-fold compared with those of the multi-height phase retrieval using *N*_holo_=2 and *N*_holo_=3, respectively (see Table 2).

The phase retrieval performance of our neural network is further demonstrated by imaging red blood cells (RBCs) in a whole blood smear. Using the reconstructed phase images of RBCs, the relative phase delay with respect to the background (where no cells are present) is calculated to reveal the phase integral per RBC (given in units of rad·μm^2^—see Supplementary Information for details), which is directly proportional to the volume of each cell, *V*. In Figure 3a, we compare the phase integral values of 127 RBCs in a given region of interest, which were calculated using the phase images of the network input, the network output, and the multi-height phase recovery image obtained with *N*_holo_=8. Due to the twin-image and other self-interference-related spatial artifacts, the effective cell volume and the phase integral values calculated using the network input image demonstrated a highly random behavior. This behavior is shown as the scattered blue dots in Figure 3a and is significantly improved by the network output, shown as the red dots in the same figure.

**Figure 4:**
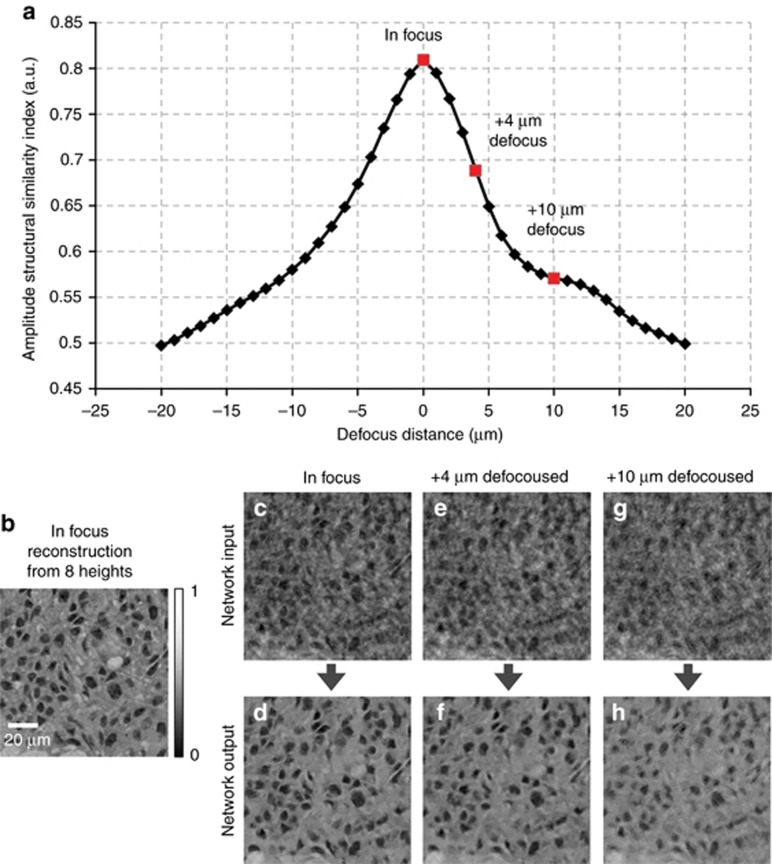
Estimation of the depth defocusing tolerance of the deep neural network. (**a**) SSIM index for the neural network output images when the input image is defocused (that is, deviates from the optimal focus used in the training of the network). The SSIM index compares the network output images in **d**, **f** and **h**, with the image obtained by using the multi-height phase recovery algorithm with *N*_holo_=8, shown in **b**.

Next, to evaluate the tolerance of the deep neural network and its holographic reconstruction framework to axial defocusing, we digitally back-propagated the hologram intensity of a breast tissue section to different depths, that is, defocusing distances within a range of *z*=[−20 μm, +20 μm] with Δ*z*=1 μm increments. After this defocusing, we then fed each resulting complex-valued image as input into the same fixed neural network, which was trained by using in-focus images at *z*=0 μm. The amplitude SSIM index of each network output was evaluated with respect to the multi-height phase recovery image with *N*_holo_=8 used as the reference (Figure 4). Although the deep neural network was trained with in-focus images, Figure 4 demonstrates the ability of the network to blindly reconstruct defocused holographic images with a negligible drop in image quality across the imaging system’s depth of field, which is ~4 μm.

In a digital in-line hologram, the intensity of the light incident on the sensor array can be written as


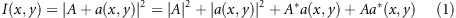


where *A* is the uniform reference wave that is directly transmitted and *a*(*x*,*y*) is the complex-valued light wave that is scattered by the sample. Under plane wave illumination, we can assume that *A* has zero phase at the detection plane, without loss of generality, that is, *A*=|*A*|. For a weakly scattering object, the self-interference term |*a*(*x*,*y*)|^2^ can be ignored compared with the other terms in Equation (1) because 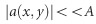. As detailed in our Supplementary Information, none of the samples that we imaged in this work satisfies this weakly scattering assumption. More specifically, the root-mean-squared (RMS) modulus of the scattered wave was measured to be approximately 28%, 34% and 37% of the reference wave RMS modulus for breast tissue, Pap smear and blood smear samples, respectively. This is why, for in-line holographic imaging of such strongly scattering and structurally dense samples, self-interference-related terms, in addition to twin-image terms, form strong image artifacts in both the phase and amplitude channels of the sample, making it difficult to apply object support-based constraints for phase retrieval. This necessitates additional holographic measurements for traditional phase recovery and holographic image reconstruction methods such as the multi-height phase recovery approach that we used for comparison in this work. Without increasing the number of holographic measurements, our deep neural network-based phase retrieval technique can learn to separate/clean the phase and amplitude images of the objects from twin-image and self-interference-related spatial artifacts, as illustrated in Figures 1, 2, 3. In principle, one could also use off-axis interferometry^55, 56, 57^ to image strongly scattering samples. However, this would create a penalty in the resolution or field of view of the reconstructed images due to the reduction in the space-bandwidth product of an off-axis imaging system.

Another important property of this deep neural network-based holographic reconstruction framework is that it significantly suppresses out-of-focus interference artifacts, which frequently appear in holographic images due to dust particles or other imperfections in various surfaces or optical components of the imaging setup. These naturally occurring artifacts are also highlighted in Figure 2f, 2g, 2n, 2o with yellow arrows and cleaned in the corresponding network output images of Figure 2d, 2e, 2l, 2m. From the perspective of our trained neural network, this property to suppress out-of-focus interference artifacts stems from the fact that these holographic artifacts fall into the same category as twin-image artifacts due to the spatial defocusing operation, helping the trained network reject such artifacts in the reconstruction process. This is especially important for coherent imaging systems because various unwanted particles and features form holographic fringes on the sensor plane and superimpose on the object’s hologram, degrading the perceived image quality after image reconstruction.

In this study, we used the same neural network architecture depicted in Figure 1 and Supplementary Figs. S1–S2 for all object types, and based on this design, we separately trained the convolutional neural network for different types of objects (for example, breast tissue vs Pap smear). The neural network was then fixed after the training process to blindly reconstruct the phase and amplitude images of any object of the same type. If a different type of sample (for example, a blood smear image) was used as an input for a specific network trained on a different sample type (for example, Pap smear images), reconstruction artifacts would appear, as exemplified in Supplementary Fig. S6. However, this does not pose a limitation because in most imaging experiments, the type of the sample is known, although its microscopic features are unknown and must be revealed with a microscope. This is the case for biomedical imaging and pathology since the samples are prepared (for example, stained and fixed) with the correct procedures, tailored for the type of the sample. Therefore, the use of an appropriately trained neural network for a given type of sample can be considered well aligned with traditional uses of digital microscopy tools.

**Figure 5:**
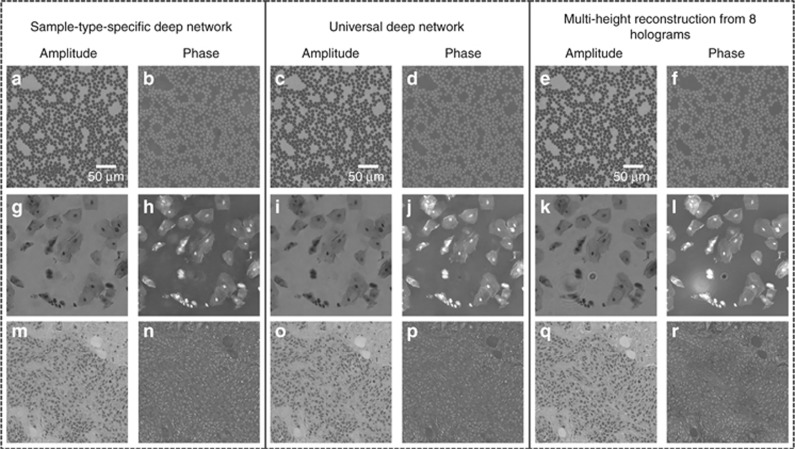
Comparison of the holographic image reconstruction results for the sample-type-specific and universal deep networks for different types of samples. The deep neural network used a single hologram intensity as input, whereas *N*_holo_=8 was used in the column on the right. (**a**–**f**) Blood smear. (**g**–**l**) Papanicolaou smear. (**m**–**r**) Breast tissue section.

We also created and tested a universal neural network that can reconstruct different types of objects after its training, based on the same architecture used in our earlier networks. To handle different object types using a single neural network, we increased the number of feature maps in each convolutional layer from 16 to 32 (Supplementary Information), which also increased the complexity of the network, leading to increased training times. However, the reconstruction runtime (after the network was fixed) increased marginally from approximately 6.45 s to 7.85 s for a field of view of 1 mm^2^ (Table 2). Table 1 also compares the SSIM index values achieved using this universal network, which performed similarly to the individual object-type-specific networks. A further comparison between the holographic image reconstructions achieved by this universal network and the object-type-specific networks is also provided in Figure 5, confirming the same conclusion as in Table 1.

## Conclusions

In this paper, we demonstrated that a convolutional neural network can perform phase recovery and holographic image reconstruction after training. This deep learning-based technique provides a new framework in holographic image reconstruction by rapidly eliminating twin-image and self-interference-related artifacts using only one hologram intensity. Compared to existing holographic phase recovery approaches, this neural network framework is significantly faster to compute and reconstructs improved phase and amplitude images of the objects with less number of measurements.

## Materials and methods

### Multi-height phase recovery

To generate the ground truth amplitude and phase images used to train the neural network, phase retrieval was achieved by using a multi-height phase recovery method^19, 21, 22^. For this purpose, the image sensor is shifted in the *z* direction away from the sample by ~15 μm increments 6 times and ~90 μm increment once, resulting in 8 different relative *z* positions of approximately 0, 15, 30, 45, 60, 75, 90 and 180 μm. We refer to these positions as the 1st, 2nd, …, 8th heights, respectively. The holograms at the 1st, 7th and 8th heights are used to initially calculate the optical phase at the 7th height, using the transport of intensity equation (TIE) through an elliptic equation solver^52^ implemented in MATLAB (Release R2016b, The MathWorks, Inc., Natick, MA, USA). Combined with the square root of the hologram intensity acquired at the 7th height, the resulting complex field is used as an initial guess for the subsequent iterations of the multi-height phase recovery. This initial guess is digitally refocused to the 8th height, where the amplitude of the guess is averaged with the square root of the hologram intensity acquired at the 8th height, and the phase information is kept unchanged. This updating procedure is repeated at the 7th, 6th,..., 1st heights, which defines one iteration of the algorithm. Usually, 10–20 iterations give satisfactory reconstruction results. However, to ensure the optimality of the phase retrieval for the training of the network, the algorithm is iterated 50 times, after which the complex field is back-propagated to the sample plane, yielding the amplitude and phase or real and imaginary images of the sample. These resulting complex-valued images are used to train the network and provide comparison images for the blind testing of the network output.

### Generation of training data

To generate the training data for the deep neural network, each resulting complex-valued object image from the multi-height phase recovery algorithm, as well as the corresponding single hologram back-propagation image (which includes the twin-image and self-interference-related spatial artifacts), is divided into 5 × 5 sub-tiles with an overlap of 400 pixels in each dimension. For each sample type, this results in a dataset of 150 image pairs (that is, complex-valued input images for the network and the corresponding multi-height reconstruction images), which are divided into 100 image pairs for training, 25 image pairs for validation, and 25 image pairs for blind testing. The average computation time for the training of each sample-type-specific deep neural network (which is done only once) was ~14.5 h, whereas it increased to approximately 22 h for the universal deep neural network (refer to Supplementary Information for additional details). As an example, the progression of the universal network training as a function of the number of epochs is shown in Supplementary Fig. S4.

### Speeding up holographic image reconstruction using GPU programming

As further detailed in the Supplementary Information, the pixel super-resolution and multi-height phase retrieval algorithms are implemented in C/C++ and accelerated using the CUDA Application Program Interface (API). These algorithms are run on a laptop computer using a single NVIDIA (Santa Clara, California) GTX 1080 graphics card. The basic image operations are implemented using customized kernel functions and are tuned to optimize the GPU memory access based on the access patterns of individual operations. GPU-accelerated libraries, such as cuFFT^58^ and Thrust^59^, are utilized for development productivity and optimized performance. The TIE initial guess is generated using a MATLAB-based implementation, which is interfaced using the MATLAB C++ engine API, allowing the overall algorithm to be maintained within a single executable after compilation.

### Sample preparation

#### *Breast tissue slide*

Formalin-fixed paraffin-embedded (FFPE) breast tissue is sectioned into 2 μm slices and stained using hematoxylin and eosin (H&E). The de-identified and existing slides are obtained from the Translational Pathology Core Laboratory at UCLA.

#### *Pap smear*

De-identified and existing Papanicolaou smear slides were obtained from the UCLA Department of Pathology.

#### *Blood smear*

De-identified blood smear slides are purchased from Carolina Biological (Item # 313158).

## Figures and Tables

**Table 1:** Comparison of the SSIM index values between the deep neural network output images obtained with a single hologram intensity (for both the sample-type-specific (STS) and universal networks) and the multi-height phase retrieval results for different numbers of input holograms (*N*_holo_) corresponding to Pap smear samples, breast tissue histopathology slides and blood smear samples

Reconstruction method	Deep network input (*N*_holo_=1)	Deep network output (STS) (*N*_holo_=1)	Deep network output (Universal) (*N*_holo_=1)	Multi-height phase-recovery (*N*_holo_=2)	Multi-height phase-recovery (*N*_holo_=3)	Multi-height phase-recovery (*N*_holo_=4)	Multi-height phase-recovery (*N*_holo_=5)	Multi-height phase-recovery (*N*_holo_=6)	Multi-height phase-recovery (*N*_holo_=7)	Multi-height phase-recovery (*N*_holo_=8)
Sample type										
Pap smear real part	0.726	0.895	0.893	0.875	0.922	0.954	0.979	0.985	0.986	1
Pap smear imaginary part	0.431	0.870	0.870	0.840	0.900	0.948	0.979	0.986	0.987	1
Blood smear real part	0.701	0.942	0.951	0.890	0.942	0.962	0.970	0.975	0.977	1
Blood smear imaginary part	0.048	0.930	0.925	0.46	0.849	0.907	0.935	0.938	0.955	1
Breast tissue real part	0.826	0.916	0.921	0.931	0.955	0.975	0.981	0.983	0.984	1
Breast tissue imaginary part	0.428	0.912	0.916	0.911	0.943	0.970	0.979	0.981	0.982	1

In each case, the SSIM index is separately calculated for the real and imaginary parts of the resulting complex-valued image with respect to the multi-height phase recovery result for *N*_holo_=8, and thus, by definition, the last column on the right has an SSIM index of 1. Due to the presence of twin-image and self-interference artifacts, the first column formed by the input images has the worst performance.

**Table 2:** Comparison of the holographic image reconstruction runtime for a field of view of ~1 mm^2^ for different phase recovery approaches

	Deep network output (STS) (*N*_holo_=1)	Deep network output (Universal) (*N*_holo_=1)	Multi-height phase-recovery (*N*_holo_=2)	Multi-height phase-recovery (*N*_holo_=3)	Multi-height phase-recovery (*N*_holo_=4)	Multi-height phase-recovery (*N*_holo_=5)	Multi-height phase-recovery (*N*_holo_=6)	Multi-height phase-recovery (*N*_holo_=7)	Multi-height phase-recovery (*N*_holo_=8)
Runtime (s)	6.45	7.85	23.20	28.32	32.11	35.89	38.28	43.13	47.43

All the reconstructions were performed on a laptop using a single GPU (see Supplementary Information for details). Of the 6.45 s and 7.85 s required for image reconstruction from a single hologram intensity using sample-type-specific and universal neural networks, respectively, the deep neural network processing time is 3.11 s for the sample-type-specific network and 4.51 s for the universal network, while the rest of the time (that is, 3.34 s for the preprocessing stages) is used for other operations such as pixel super-resolution, auto-focusing and free space back-propagation.
