# Supplementary material for: Phase recovery and holographic image reconstruction using deep learning in neural networks
Source: Light Sci Appl. 2018 Feb 23;7:17141–. doi: 10.1038/lsa.2017.141 (PMC6060068; doi:10.1038/lsa.2017.141)
Supplement: Supplementary Information [file lsa2017141x1.pdf]

Supplementary Information

Phase recovery and holographic image reconstruction using deep learning in neural networks

Yair Rivenson[1,2,3]†, Yibo Zhang[1,2,3]†, Harun Günaydın[1], Da Teng[1,4], Aydogan Ozcan[1,2,3,5]*

[1]*Electrical Engineering Department, University of California, Los Angeles, CA, 90095, USA.*

[2]*Bioengineering Department, University of California, Los Angeles, CA, 90095, USA.*

[3]*California NanoSystems Institute (CNSI), University of California, Los Angeles, CA, 90095, USA.*

[4]*Computer Science Department, University of California, Los Angeles, CA, 90095, USA*

[5]*Department of Surgery, David Geffen School of Medicine, University of California, Los Angeles, CA, 90095, USA.*

*Correspondence: ozcan@ucla.edu

Network architecture

Our deep neural network architecture is detailed in Fig. 1 and Supplementary Figs. 1-2. The real and imaginary parts of the back-propagated hologram intensity are used as two input image channels to the network, each with a size of $M\times N$ pixels (e.g., $M = 1392$, $N = 1392$). Although not considered here, in an alternative implementation a different network design could possibly use the raw hologram intensity as input, without a wave back-propagation step. These two channels (real and imaginary parts) of the network are then used *simultaneously* as input to 4 convolutional layers. The output of each convolutional layer is 16 channels (feature maps), each with a size of $M\times N$ pixels, which was empirically determined to balance the deep network size/compactness and performance. The value of x,y-th pixel in the j-th feature map in the i-th convolutional layer is given by $v_{i,j}^{x,y}$:[1]

$$v_{i,j}^{x,y} = \sum_r \sum_{p=0}^{P-1} \sum_{q=0}^{Q-1} w_{i,j,r}^{p,q} v_{i-1,r}^{x+p,y+q} + b_{i,j} \quad \text{(s1)}$$

where $b_{i,j}$ is a common bias term for the j-th feature map, r indicates the set of the feature maps in the i-1 layer (which is 2, for the first convolutional layer), $w_{i,j,r}^{p,q}$ is the value of the convolution kernel at the p,q-th position, P and Q define the size of the convolutional kernels, which is 3×3 throughout the network in our implementation.

For object type-based deep networks, the output of these 4 convolutional layers is then downsampled by ×1, ×2, ×4, ×8, creating 4 different data flow paths, with 16 channels and spatial dimensions of $M\times N$, $M/2\times N/2$, $M/4\times N/4$ and $M/8\times N/8$, respectively. This multi-scale data processing scheme was created to allow the network to learn how to suppress the twin-image and self-interference artifacts, created by objects with different feature sizes. The output of these downsampling operators is followed by 4 residual blocks[2], each composed of 2 convolutional layers and 2 activation functions, which we chose to implement as rectified linear units (ReLU), i.e., $\mathrm{ReLU}(x) = \max(0, x)$. Residual blocks create a shortcut between the block's input and output, which allows a clear path for information flow between layers[3]. This has been demonstrated to speed up the convergence of the training phase of the deep neural network. While some of the previously reported residual block architectures contain batch normalization layers, we observed that the addition of such layers into our network reduces the inference performance and the quality of the phase retrieval results, and therefore we did not use batch normalization in our architecture. Following the 4 residual blocks, data at each scale are upsampled to match the original data dimensions. Each upsampling block[4] (i.e., U/S block in Supplementary Fig. 1) contains a convolutional layer that takes 16 channels, each with $M/L\times N/L$ pixels as input, and outputs 64 channels each with $M/L\times N/L$ pixels (L=2, 4, 8). This is followed by a ReLU operation and an upsampling layer, which is schematically detailed in Supplementary Fig. 2. This layer learns to upsample a 64 channel input (each with $M/L\times N/L$ pixels) to a 16 channel output (each with $2M/L\times 2N/L$ pixels). This upsampling process is being performed once, twice, or three times, for the ×2, ×4, ×8 spatially downsampled network inputs, respectively (see Supplementary Fig. 1). The output of each one of these 4 different dataflow paths (with 16 channels, $M\times N$ pixels, following the upsampling stage) is concatenated to a 64 channels input, which results in 2 channels: one for the real part and one for the imaginary part of the object image, each having $M\times N$ pixels. For the universal deep network, we kept the same architecture;

however, we increased the number of channels in the output of each convolutional layer by two-fold, i.e., from 16 to 32 in the residual blocks.

To train the network, we minimized the average of the mean-squared-errors of the real and imaginary parts of the network output with respect to the real and imaginary parts of the object's ground truth images, obtained using multi-height phase retrieval with 8 holograms recorded at different sample-to-sensor distances (also see the Methods section of the main text). This loss function over a mini-batch of K input patches (images) is calculated as:

$$Loss(\Theta) = \frac{1}{2K}\sum_{k=1}^{K}\left\{\frac{1}{M\times N}\sum_{m=1}^{M}\sum_{n=1}^{N}\left\|Y_{\mathrm{Re},m,n,k}^{\Theta} - Y_{\mathrm{Re},m,n,k}^{GT}\right\|^2 + \frac{1}{M\times N}\sum_{m=1}^{M}\sum_{n=1}^{N}\left\|Y_{\mathrm{Im},m,n,k}^{\Theta} - Y_{\mathrm{Im},m,n,k}^{GT}\right\|^2\right\} \quad \text{(s2)}$$

where k is the k-th image patch, $Y_{\mathrm{Re},m,n,k}^{\Theta}$, $Y_{\mathrm{Im},m,n,k}^{\Theta}$ denote the m,n-th pixel of real and imaginary network outputs, respectively, and $Y_{\mathrm{Re},m,n,k}^{GT}$, $Y_{\mathrm{Im},m,n,k}^{GT}$ denote the m,n-th pixel of real and imaginary parts of the training (i.e., ground truth) labels, respectively. The network's parameter space (e.g., kernels, biases, weights) is defined by Θ and its output is given by $[Y_{\mathrm{Re}}^{\Theta}, Y_{\mathrm{Im}}^{\Theta}] = F(X_{\mathrm{Re},input}, X_{\mathrm{Im},input};\Theta)$, where F defines the deep neural network's operator on the back propagated complex field generated from a single hologram intensity, divided into real and imaginary channels, $X_{\mathrm{Re},input}, X_{\mathrm{Im},input}$, respectively. Following the estimation of the loss function, the resulting error in the network output is back-propagated through the network and the Adaptive Moment Estimation[5] (ADAM) based optimization is used to tune the network's parameter space, Θ, with a learning rate of 10^{-4}. For the sample type specific network training, we used a batch size of K=2 and an image size of 1392×1392 pixels. For the universal deep network, we divided the image dataset to 256×256-pixel patches (with an overlap of 20% between the patches) and a mini-batch size of K=30 (see Supplementary Fig. 3). For both the sample type specific and universal networks, the images that we used to test the network had a size of 1392×1392 pixels. All the convolutional kernel entries are initialized using a truncated normal distribution. All the network bias terms, $b_{i,j}$, are initialized to 0. In case the size of the input image is not divisible by 8, zero padding is performed on it such that it becomes divisible by 8.

As an example, the progression of the universal deep network training is shown Supplementary Fig. 4 by plotting the training and validation dataset errors as a function of the number of training epochs (i.e., the number of passes on the entire dataset with backpropagation through the network).

Network implementation details

For our programming, we used Python version 3.5.2, and the deep neural network was implemented using TensorFlow framework version 1.1.0 (Google). We used a laptop computer with Core i7-6700K CPU @ 4GHz (Intel) and 64GB of RAM, running a Windows 10 operating system (Microsoft). The network training was performed using GeForce GTX 1080 (Nvidia) Dual Graphical Processing Units (GPUs). The testing of the network was performed on *a single GPU* to provide a fair comparison against multi-height phase retrieval CUDA implementation, as summarized in Table 2 (main text).

Optical set-up

Our experimental set-up (Supplementary Fig. 5) includes a laser source (SC400, Fianium Ltd., Southampton, UK) filtered by an acousto-optic tunable filter and coupled to a single mode optical fiber to provide partially coherent illumination with a spectral bandwidth of ~2.5 nm. A CMOS image sensor with 1.12 μm pixel size and 16.4 Megapixel (IMX081, Sony Corp., Japan) is used to capture the holographic images. The distance from the optical fiber tip to the sample is between 7 and 15 cm, such that the light that is incident on the sample can be considered a quasi-plane wave. The distance from the sample to the image sensor plane is approximately 300-700 μm. This unit magnification geometry results in a large field of view that is equal to the image sensor's active area. The image sensor was mounted on a 3D positioning stage (NanoMax 606, Thorlabs Inc., New Jersey, US), which moved it in *x* and *y* directions in sub-pixel-size steps to implement pixel super-resolution (PSR). The image sensor was also shifted in the z direction with step sizes of a few tens of microns to perform multi-height phase recovery to generate training data for the neural network. A custom-written LabVIEW program implemented on a desktop computer was used to control and automate all of these components as part of the imaging set-up.

Pixel super resolution (PSR)

In order to mitigate the spatial undersampling caused by the relatively large pixel pitch of the image sensor chip (~1.12 μm), multiple subpixel-shifted holograms were used to synthesize a higher resolution (i.e., pixel super-resolved) hologram. For this, the image sensor was mechanically shifted by a 6-by-6 rectangular grid pattern in the x-y plane, with increments of 0.37 μm, corresponding to approximately 1/3 of the image sensor's pixel size. A 6-by-6 grid ensured that one color channel of the Bayer pattern could cover its entire period. In an alternative design with a monochrome image sensor (instead of an RGB sensor), only a 3-by-3 grid would be needed to achieve the same PSR factor. For this PSR computation, an efficient non-iterative fusion algorithm was applied to combine these sub-pixel shifted images into one higher-resolution hologram, which preserves the optimality of the solution in the maximum likelihood sense[6]. The selection of which color channel (R, G or B) of the Bayer pattern to use for holographic imaging is based on pixel sensitivity to the illumination wavelength that is used. For example, at ~530 nm illumination, the two green channels of the Bayer pattern were used, and at ~630 nm, the red channel was used.

Calculation of red blood cell (RBC) phase integral and effective refractive volume

The relative optical phase delay due to a cell, with respect to the background, can be approximated as:

$$\varphi(x,y) = \frac{2\pi d(x,y) \cdot \Delta n(x,y)}{\lambda} \qquad \text{(s3)}$$

where $d(x,y)$ is the thickness of the sample (e.g., an RBC) as a function of the lateral position, $\Delta n(x,y) = n(x,y) - n_0$ is the refractive index difference between the sample ($n(x,y)$) and the background medium (n_0), λ is the illumination wavelength in air. Based on these, we define the phase integral for a given RBC image as:

$$p_i = \left| \int_{S_i} \varphi(x,y)\,\mathrm{d}s \right| = \left| \int_{S_i} \frac{2\pi d(x,y)\Delta n(x,y)}{\lambda}\,\mathrm{d}s \right| \quad \text{(s4)}$$

which calculates the relative phase with respect to the background that is integrated over the area of each RBC (defined by S_i), which results in a unit of rad·μm^2. Let Δn represent the average refractive index difference within each cell (with respect to n_0), we can then write:

$$p_i = \frac{2\pi \cdot |\Delta n|}{\lambda} \int_{S_i} d(x,y) \cdot \mathrm{d}s = \frac{2\pi \cdot |\Delta n|}{\lambda} \cdot V_i \quad \text{(s5)}$$

where V_i represents the volume of the i-th cell. Because the average refractive index of a *fixed and stained* RBC (as one would have in a blood smear sample) is hard to determine or estimate, we instead define *effective refractive volume of an RBC* as:

$$\tilde{V}_i = |\Delta n| \cdot V_i = \frac{p_i \lambda}{2\pi} \quad \text{(s6)}$$

which also has the unit of volume (e.g., femtoliter, fL).

Structural similarity (SSIM) index calculation

The structural similarity index between two images I_1 and I_2 can be calculated as[7]:

$$\mathrm{SSIM}(I_1, I_2) = \frac{(2\mu_1\mu_2 + c_1)(2\sigma_{1,2} + c_2)}{(\mu_1^2 + \mu_2^2 + c_1)(\sigma_1^2 + \sigma_2^2 + c_2)} \quad \text{(s7)}$$

where μ_1 is the average of I_1, μ_2 is the average of I_2, σ_1^2 is the variance of I_1, σ_2^2 is the variance of I_2, $\sigma_{1,2}$ is the cross-covariance of I_1, and I_2. The stabilization constants (c_1, c_2) prevent division by a small denominator and can be selected as $c_1 = (K_1 L)^2$ and $c_2 = (K_2 L)^2$, where L is the dynamic range of the image and K_1, K_2 are both much smaller than 1. SSIM index between two images ranges between 0 and 1 (the latter for identical images).

Evaluation of scattering strength of the samples

To evaluate the validity of the weakly scattering condition, i.e., $|a(x,y)| \ll A$ for the samples that we imaged (see the Discussion section of the main text), we took a region of interest for each of the samples that is reconstructed using the multi-height phase recovery, based on 8 hologram heights. After the phase recovery step, we have:

$$u = A + a(x,y) \quad \text{(s8)}$$

where A can be estimated by calculating the average value of a background region where no sample is present. After A is estimated, we calculate a normalized complex image $\tilde{u}$,

$$\tilde{u} = \frac{u}{A} = 1 + \frac{a(x, y)}{A} \quad \text{(s9)}$$

Next, we define R as the ratio between the root-mean-squared (RMS, or quadratic mean) modulus of the scattered wave $|a(x,y)|$ divided by the reference wave modulus $|A|$, to obtain:

$$R = \frac{\left\langle |a(x, y)|^2 \right\rangle^{1/2}}{|A|} = \left\langle |\tilde{u} - 1|^2 \right\rangle^{1/2} \quad \text{(s10)}$$

where $\langle \bullet \rangle$ denotes 2D spatial averaging operation. This ratio, R, is used to evaluate the validity of the weakly scattering condition for our samples, and is found to be 0.28, 0.34, and 0.37 for the breast tissue, Pap smear and blood smear samples that we imaged, respectively (see the Discussion section).

Calculation of the sample-to-sensor distance

The relative separation between successive image sensor heights (or hologram planes) needs to be estimated to successfully apply the TIE and multi-height phase recovery algorithms, and the absolute z_2 distance (i.e., the sample-to-sensor distance, see Supplementary Fig. 5) is needed for the final back-propagation of the recovered complex wave onto the sample plane. Estimating the relative z-separation is done by using an autofocusing algorithm based on an axial magnitude differential metric, i.e.,

$$z_{focus} = \arg\min_{z} \left[\frac{\partial}{\partial z} \sum_{x,y} \left| U_{\mathrm{ROI}}(x, y; z) \right| \right] \quad \text{(s11)}$$

where $U_{\mathrm{ROI}}(x, y; z)$ is the propagated complex optical wavefront at a distance of z, and cropped to a certain region of interest (ROI).

For computational efficiency, first a coarse scan is done between 100 μm and 800 μm with a step size of 10 μm. Then, around the minimum that is found by this coarse scan, a golden section search algorithm[8] is applied to locate the minimum with a final precision of 0.01 μm. The absolute z_2 is refined after the convergence of the multi-height phase recovery algorithm by refocusing the phase-recovered hologram near the previously found focus point.

References

1. Ji, S., Xu, W., Yang, M. & Yu, K. 3D Convolutional Neural Networks for Human Action Recognition. *IEEE Trans. Pattern Anal. Mach. Intell.* **35,** 221–231 (2013).

2. He, K., Zhang, X., Ren, S. & Sun, J. Deep Residual Learning for Image Recognition. in 770–778 (2016).
3. He, K., Zhang, X., Ren, S. & Sun, J. Identity Mappings in Deep Residual Networks. in *Computer Vision – ECCV 2016* (eds. Leibe, B., Matas, J., Sebe, N. & Welling, M.) 630–645 (Springer International Publishing, 2016). doi:10.1007/978-3-319-46493-0_38
4. Shi, W. *et al.* Real-Time Single Image and Video Super-Resolution Using an Efficient Sub-Pixel Convolutional Neural Network. in 1874–1883 (2016).
5. Kingma, D. & Ba, J. Adam: A Method for Stochastic Optimization. in (2014).
6. Farsiu, S., Robinson, M. D., Elad, M. & Milanfar, P. Fast and Robust Multiframe Super Resolution. *IEEE Trans. Image Process.* **13,** 1327–1344 (2004).
7. Wang, Z., Bovik, A. C., Sheikh, H. R. & Simoncelli, E. P. Image Quality Assessment: From Error Visibility to Structural Similarity. *IEEE Trans. Image Process.* **13,** 600–612 (2004).
8. Press, W. H., Teukolsky, S. A., Vetterling, W. T. & Flannery, B. P. *Numerical Recipes in C (2Nd Ed.): The Art of Scientific Computing*. (Cambridge University Press, 1992).

Supplementary Figures

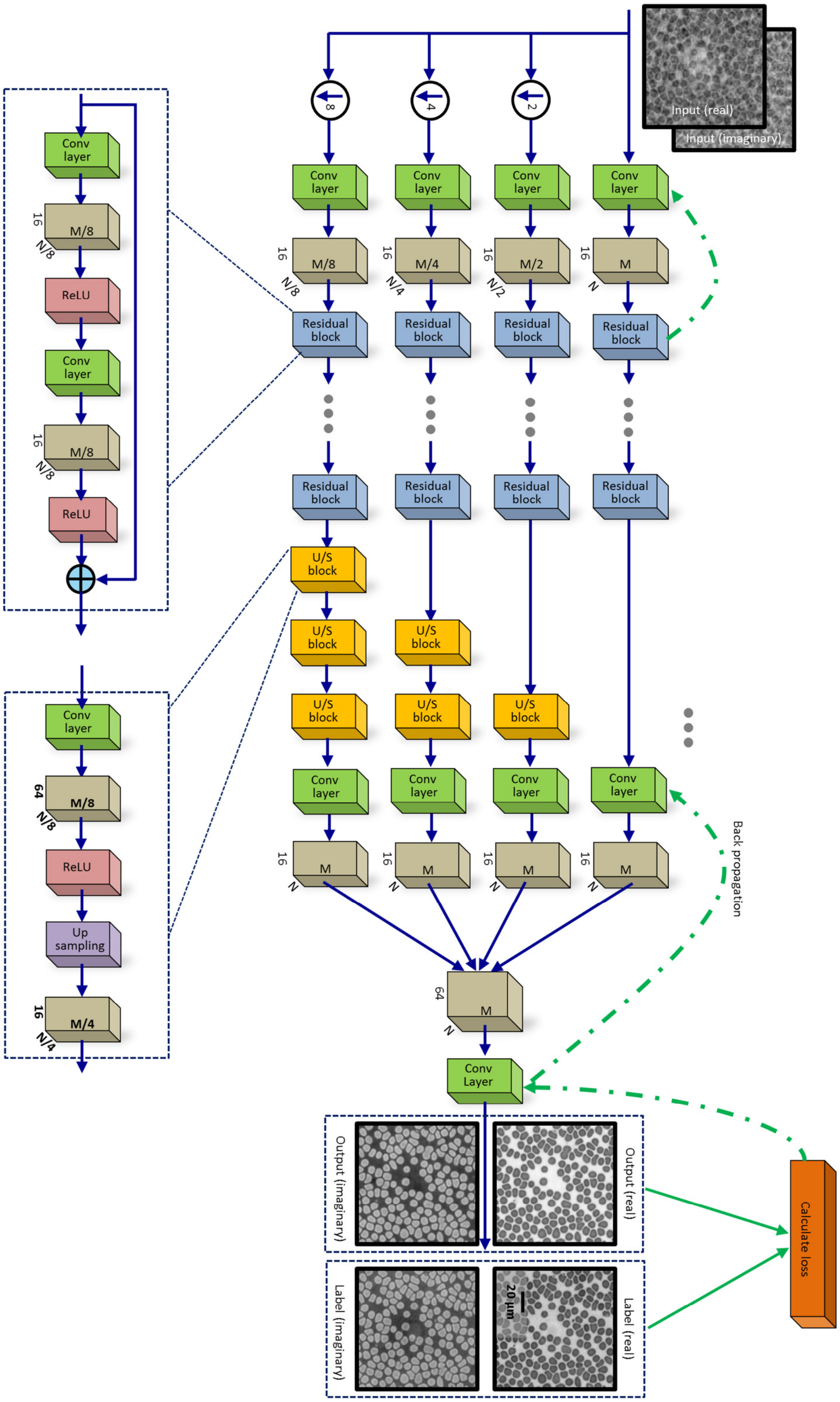

Supplementary Fig. 1. Architecture of our deep neural network and its training. The neural network is composed of convolutional layers (i.e., conv layers), upsampling blocks (U/S blocks) and nonlinear activation functions (ReLU). Also see Supplementary Fig. 2.

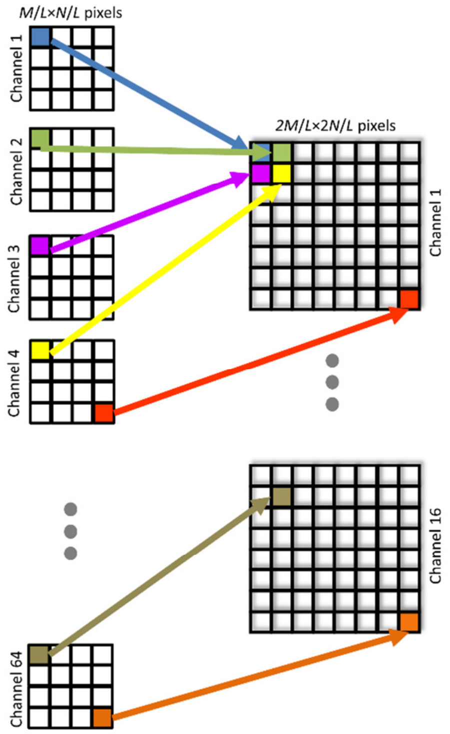

Supplementary Fig. 2. Detailed schematics of the upsampling layer of our deep neural network.

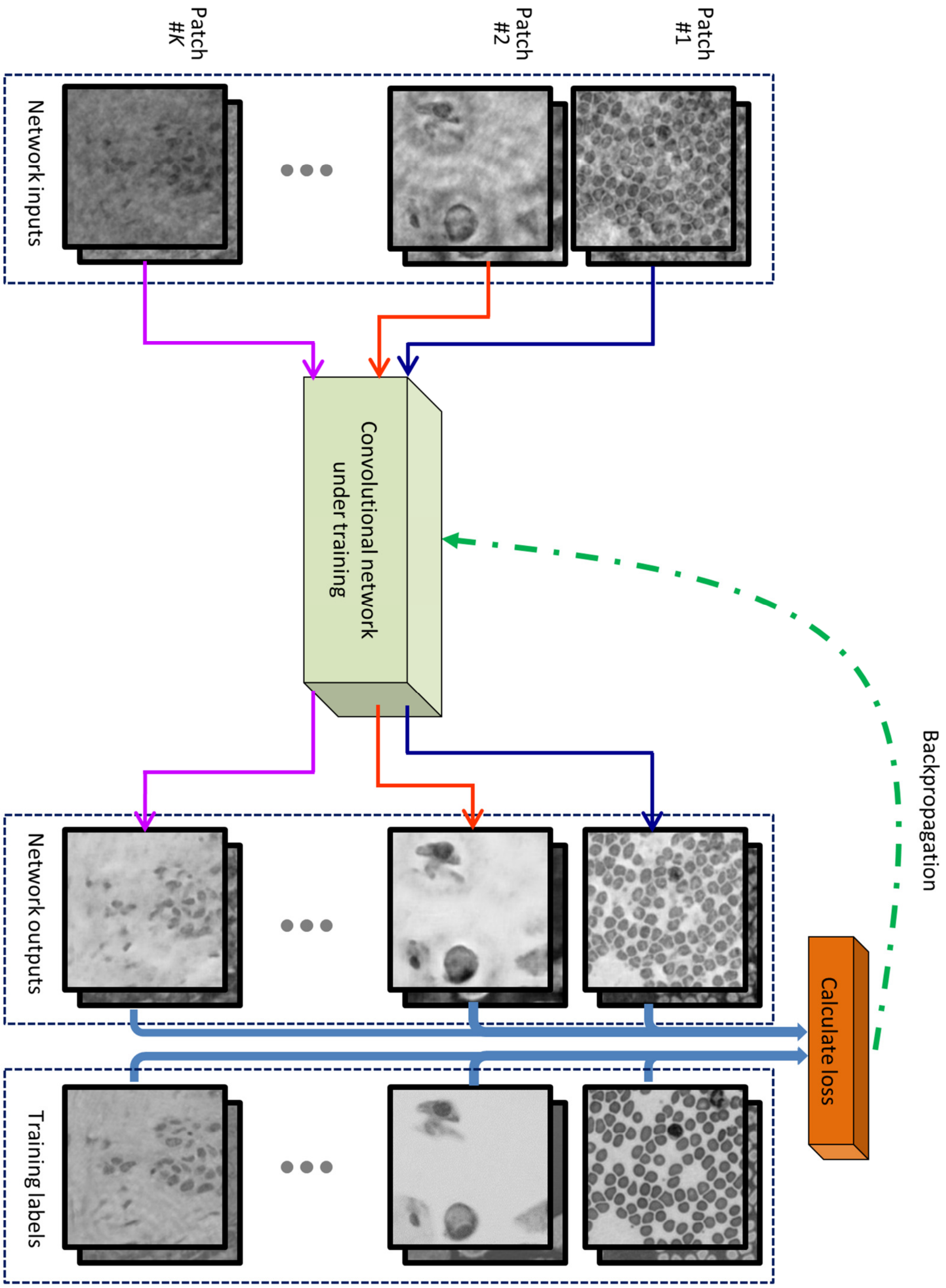

Supplementary Fig. 3. Training of the universal deep neural network that can reconstruct different types of objects.

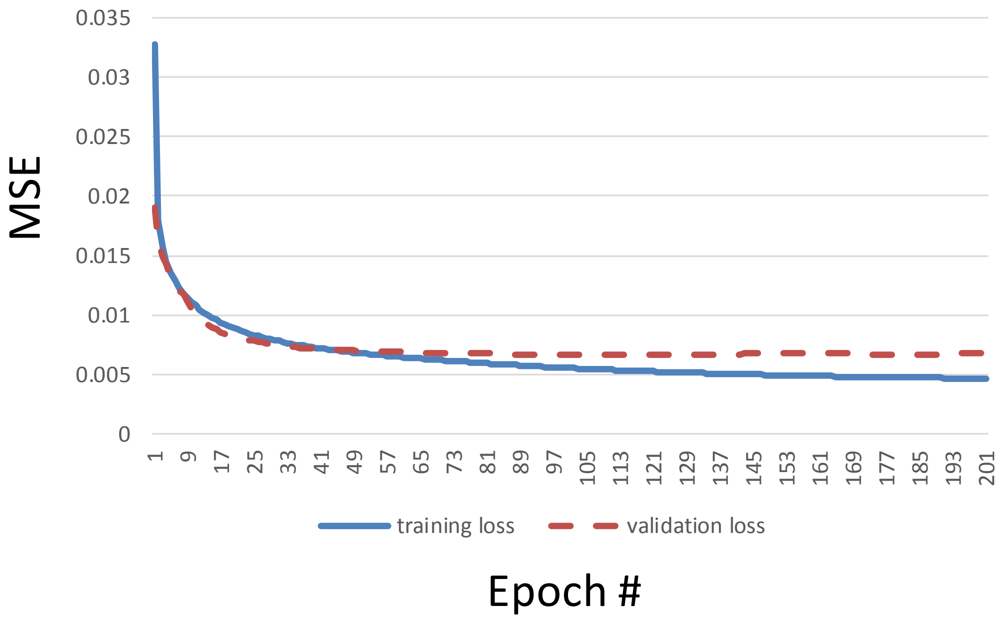

Supplementary Fig. 4. Training and validation dataset errors as a function of the number of epochs.

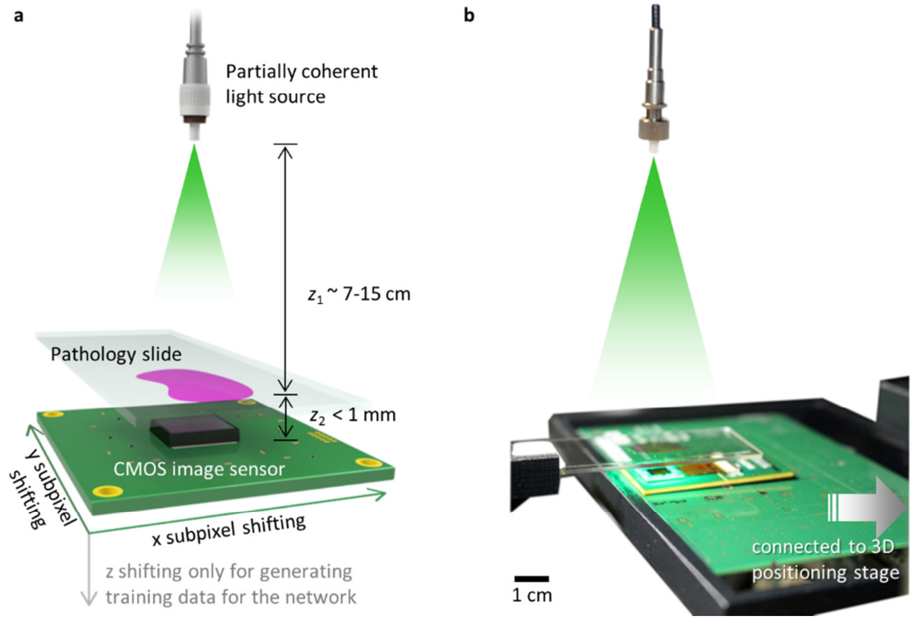

Supplementary Fig. 5. Holographic imaging setup. **a,** schematics of the optical set-up. **b,** a photograph of the same setup.

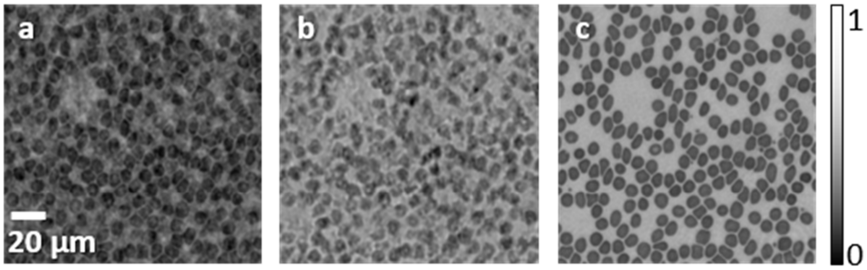

Supplementary Fig. 6. The result of feeding a blood smear hologram into a sample type specific network, which was trained with only Pap smear image data. (a) Amplitude of the input blood smear image after free space back-propagation. (b) Pap smear trained network output image (amplitude) in response to (a). (c) Image of the same sample obtained by using the multi-height phase recovery algorithm with $N_{holo} = 8$.
